# Urachal carcinoma revealed by isolated hematuria

**DOI:** 10.1016/j.amsu.2021.102193

**Published:** 2021-02-25

**Authors:** Issam Jandou, Adnan Ettanji, Ettaouil Mohammed, Amine Moataz, Dakir Mohammed, Adil Debbagh, Rachid Aboutaieb

**Affiliations:** a University Hospital Center, Ibn Rochd, Casablanca, Morocco; b Faculté de médicine et de pharmacie, Casablanca, Morocco; c Laboratoire de santé sexuelle, Faculté de médecine et de pharmacie, Université Hassan II, Casablanca, Morocco

**Keywords:** Urachus, Adenocarcinoma, Hematuria, Bladder cancer

## Abstract

Urachal carcinoma is an aggressive and rare neoplasia of bladder cancer involving the urachus. The diagnostic failure is due to its insidious development as well as its non-specific clinical signs. Management constitutes a real dilemma for urological surgeons. We describe two pathological cases of urachal adenocarcinoma revealed by isolated hematuria.

## Highlights

• Urachus cancer is a rare malignant tumor.
• The tomodensitometry is the reference examination which makes it possible to think of the diagnosis in front of a mass of urachus.
• Surgery remains the gold standard for therapeutic management.
• The prognosis is pejorative because of the aggressiveness of the tumor of the delayed diagnosis.

## 1 Introduction

Urachal adenocarcinoma is an extremely rare tumor that accounts for less than 1% of all bladder cancers [1]. It is characterized by three clues, late presentation of symptoms, early local invasion and distant metastasis which determines its poor prognosis [2]. The scarcity of cases explains the low number of studies carried out and an evidence-based management strategy is lacking.

The histogenesis is not yet well elucidated, it is based on two rather old hypotheses, the metaplastic theory and the dysplastic theory. Various recommended treatments based on surgery first [1]. The role of chemotherapy and radiotherapy is not yet well established. The prognosis of these tumors remains poor in the majority of cases. We describe two pathological cases of urachal adenocarcinoma revealed by isolated hematuria reported according to the SCARE 2020 criteria [2].

### 1.1 Case presentation

#### 1.1.1 Case 1

Mr. E.A., 46 years old, father of 6 children, the patient had a history of pulmonary tuberculosis treated 20 years ago and pericardial effusion drained 6 years ago, chronic tobacco user at 26 PA weaned 7 years ago. Symptoms go back to 4 months, marked by the appearance of terminal clot hematuria without other associated signs.

The abdominal examination is without abnormalities. The rectal examination finds a homogeneous prostate of 25 g without a suspect nodule. All evolving in a context of declining general condition (ASA2).

**Fig. 1:**
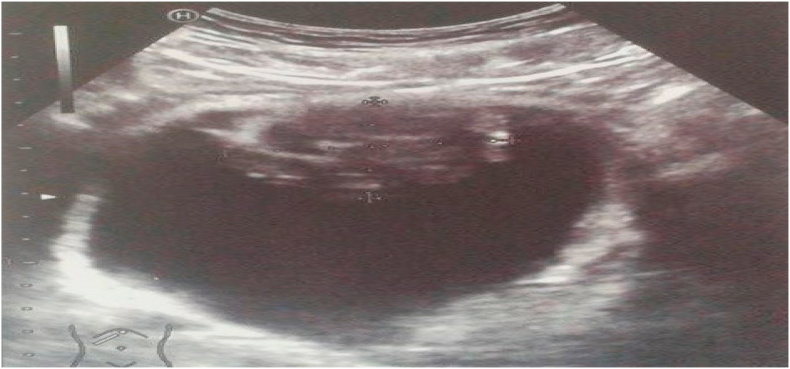
Ultrasound bladder revealed a heterogeneous tissue process budding endovesically.

Abdominal ultrasound revealed a heterogeneous tissue process budding endovesical, the site of a few cystic areas and calcifications, with irregular contours, vascularized by color Doppler. This process measuring 67.4 × 53 × 42.4mm, continues at the top and follows the path of the urachus (Fig. 1).

The patient underwent a cystoscopy which found a budding tumor with a large implantation base in the anterior wall and bladder dome. The histological study, after endoscopic resection, revealed a tumor proliferation made up of tall columnar cells, provided with a large nucleus, a fine chromatin. These cells are arranged in a villous structure, and dilated tubes filled with mucus. It is an isolated tubulovillous adenocarcinoma and infiltrating the superficial chorion of the bladder mucosa.

An immunohistochemical study which objectified an aspect compatible with a moderately differentiated CK7 positive adenocarcinoma (TTF 1 and CK20 are negative).

**Fig. 2:**
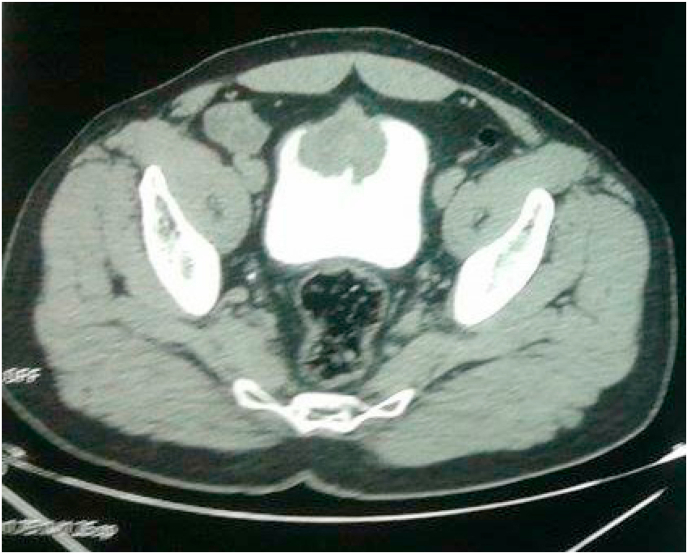
Abdomino-pelvic CT scan showed budding wall thickening of the anterior wall of the bladder.

**Fig. 3:**
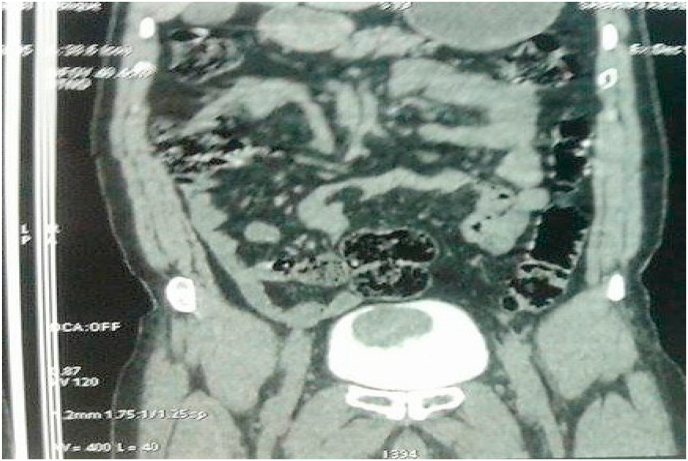
Abdomino-pelvic CT scan revealed wall thickening, budding from the anterior wall of the bladder.

Computed tomography revealed the presence of a budding wall thickening of the anterior wall of the bladder, with endo and exophytic development in the peritoneum with preservation of a fatty border of separation with the digestive loops. It measures 52 × 49 mm. He also presents in the lungs an inferior left lobe tissue process (Fig. 2, Fig. 3).

**Fig. 4:**
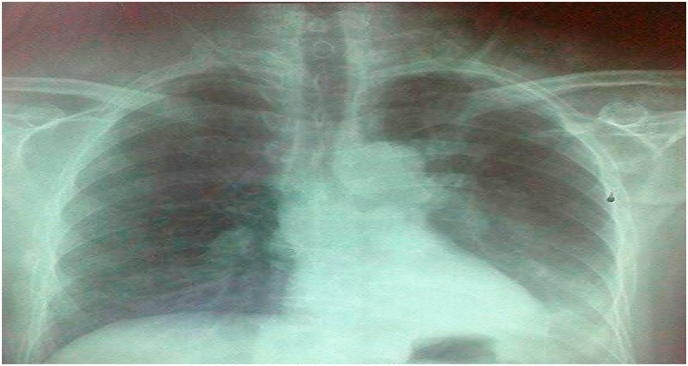
X-ray of chest face revealed opacity at the level of the left middle arch with opening of the angle of the keel.

The chest x-ray showed a left basal opacity and at the level of the left middle arch associated with a mediastinal widening with widening of the angle of the carina (Fig. 4).

The patient underwent a bronchoscopy which showed a budding tumor located at the bottom of the left main bronchus with thickened keel. The bronchial biopsy discovers the presence of glandular-looking carcinomatous masses, concluding in a bronchial localization of a glandular-like carcinoma suggesting a secondary localization of a urachal adenocarcinoma.

After multidisciplinary consultation, it was decided to treat the patient with chemotherapy alone due to the metastatic aspect. He received three courses of Folfox-type chemotherapy. Fifteen days later, he presented aplastic anemia with grade 3 mucositis and oral candidiasis.

The patient received medical treatment, rehydration, and increased monitoring for the risk of infection. After his discharge, the patient was lost to follow-up.

#### 1.1.2 Case 2

Mr. L.M, 52 years old, 30 pack-year smoking history. The symptoms go back to 1 month marked by the appearance of a terminal clotting hematuria without other urinary disorders, all evolving in a context of preservation of the general condition.

The physical examination found hypogastric pain without a palpable mass, the lumbar fossae were free, The digital rectal examination found a firm prostate of 20 g, without suspicious nodules and the remainder of the clinical examination was unremarkable.

The abdominal ultrasound revealed a tumor mass of the anterior wall of the bladder associated with dilation of the intestinal loops.

The cystoscopy found a budding tumor of 4 cm located at the level of the bladder dome. The histological study, after endoscopic resection, shows a bladder localization of a colloid mucous adenocarcinoma infiltrating the muscularis (Detrusor): pT2 at least.

An immunohistochemical study was carried out to discuss the primary or secondary nature of this tumor, in particular CK7, CK20, PSA, and CDX2, this analysis revealed an immunohistochemical aspect compatible with a bladder localization of an adenocarcinoma of digestive origin (CK20 and CDX2 are positive, CK7 and PSA are negative).

**Fig. 5:**
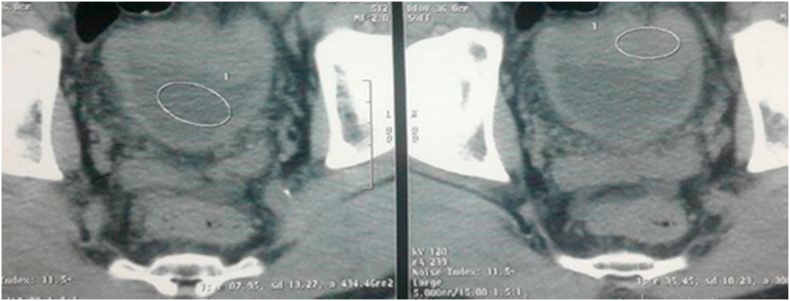
Computed tomography showed circumferential infiltration of the anterior bladder wall related to a tumor of the urachus.

Computed tomography showed a circumferential infiltration of the anterior bladder wall related to a tumor of the urachus (Fig. 5). The rectosigmoidoscopy looking for a neighboring tumor was performed twice and did not reveal anything in particular. The chest x-ray was normal.

The patient refused the surgical treatment proposed by the oncology-urology committee, which consisted of a cystectomy. Six months later, the patient was admitted for consultation with a deterioration in general condition.

**Fig. 6:**
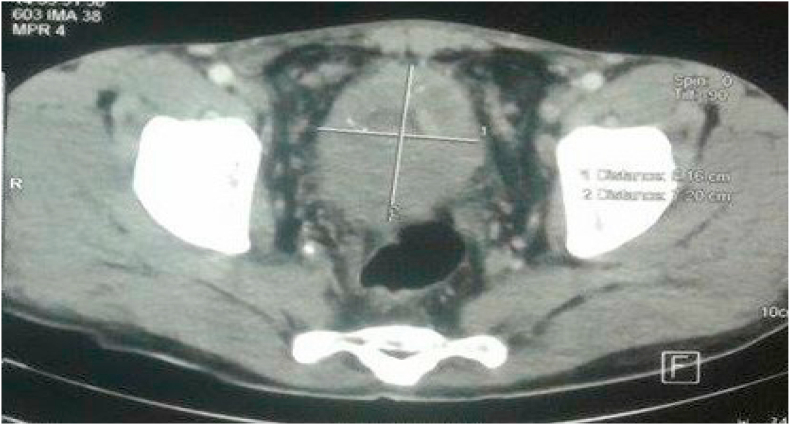
Abdomino-pelvic CT scan demonstrated a large perivesical process with endo and exo-vesical development related to the urachus tumor.

Computed tomography showed a voluminous perivesical process with endo and exo-vesical development, related to the urachus tumor measuring 72 × 61mm, arriving in contact with the umbilicus, of heterogeneous density with area of necrosis, site of microcalcifications central and peripheral (Fig. 6).

**Fig. 7:**
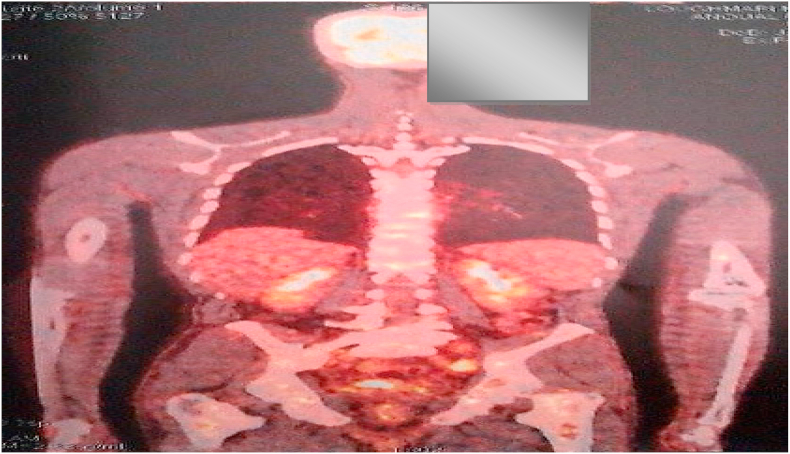
PET-SCAN showed a partly calcified hypermetabolic tumor process located in the pre-bladder space. Associated with mediastinal lymph nodes, diffuse bone lesions, abdominal and pelvic lymph nodes.

**Fig. 8:**
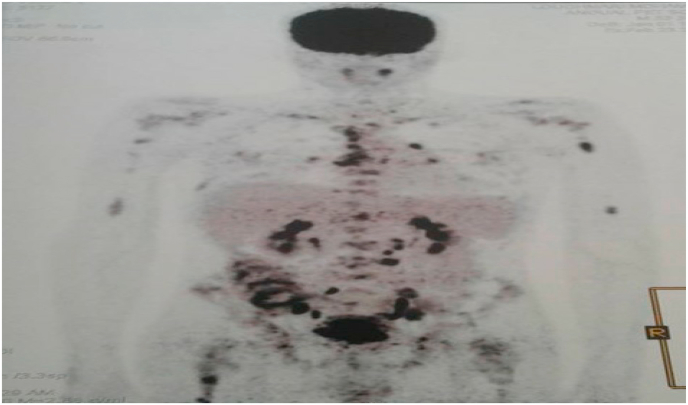
PET-SCAN revealed a partially calcified hypermetabolic tumor process located in the retropubic space. Associated with mediastinal lymph nodes, diffuse bone lesions, abdominal and pelvic lymph nodes.

**Fig. 9:**
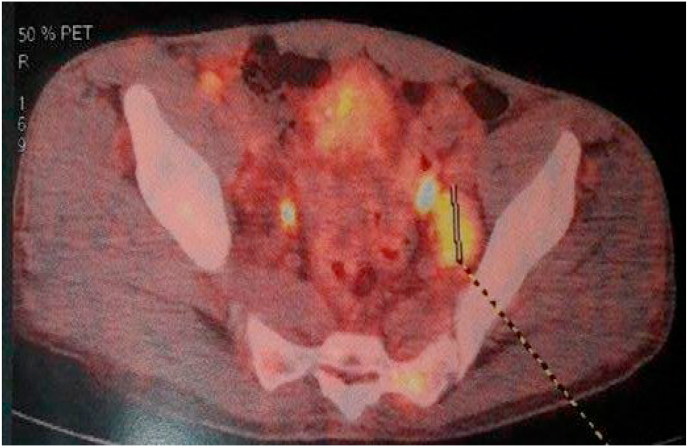
PET-SCAN objectified hypermetabolic lesions inside the left psoas, suggesting a large lymphadenopathy.

PET-SCAN demonstrated a hypermetabolic partially calcified tumor process located in the pre-bladder space. Associated with mediastinal lymph nodes, diffuse bone lesions, and abdominal and pelvic lymph nodes (Fig. 7, Fig. 8, Fig. 9).

After a multidisciplinary consultation meeting, it was decided to start palliative chemotherapy based on capecitabine 1500 mg/day in three doses per day for 15 days. After a week of treatment, the patient developed eating disorders with dizziness, vomiting, and diffuse bone pain. Analgesic rehabilitation and an adequate nutritional regime have been implemented for better support. An evaluation at 3 months shows a good tolerance to the chemotherapy.

## 2 Discussion

Urachal adenocarcinoma is an extremely rare tumor that accounts for less than 1% of all bladder cancers [1]. The incidence of urachal adenocarcinoma is estimated to be 0.01% of all cancers in adults. It accounts for 0.5–2% of all bladder tumors and 20–40% of primary bladder adenocarcinomas [3,4].

The majority of urachal tumors 93% are adenocarcinomas, and 48% produce mucin [[5], [6], [7]]. More than half of patients with adenocarcinoma of urachus are over 50 years old (58%) [6]. In previous studies, the average age of diagnosis ranged from 50 to 58 years [[8], [9], [10]].

For JOHNSON, who presented the largest world series in his time, it is difficult to incriminate the role of intra-vesical carcinogens (tobacco, chemical agents of paint such as arylamines, cyclophosphamide, etc.), because even if the urachus was permeable, the urinary reflux inside seems unlikely because of its narrow caliber and the presence of epithelial secretions which obstruct the lumen.

Because urachal cancer is poorly symptomatic, patients often present at diagnosis with a more advanced stage and a poor prognosis. The clinical signs are generally determined by the location, volume and possible extension to neighboring organs. Hematuria is the main symptom, it occurs in 90% of patients but is rarely isolated.

Voiding disturbances may manifest as signs of bladder irritation such as urination burns, urge, or pollakiuria. They reflect bladder irritation secondary to parietal invasion and the growth of the tumor above the bladder, which interferes with bladder dynamics. Obstructive signs such as dysuria can also be seen and are rather due to infiltration of the bladder wall.

The emission of mucus in the urine is rare and is only present in 25% of cases, this mucus secretion when it exists is highly suggestive of a mucosecreting adenocarcinoma (but not specific to urachal).

Hypogastric pain can be present in patients, like suprapubic pain, which was the case in only one patient in our study. A sensitive suprapubic mass can be found clinically in 25% of cases. The umbilical aspect such as an umbilical deformity with emission of pus, or blood can be revealing according to the Descazeaud study. The metastases were reported by VERGOS in a case of urachal adenocarcinoma revealed by skin metastases, The incidental finding is usually given the clinical latency [11].

The bladder ultrasound specifies the ultrasound criteria of the median mass, makes it possible to detect bladder invasion and to look for possible metastatic locations.

Computed tomography makes it possible to better visualize the extra-bladder part of the tumor, to assess the possible extension to neighboring organs, and to look for lymph node or metastatic involvement. Calcifications are found in 5–10% of these tumors [12].

Regarding the tumor site, the major part of the tumor can be seen outside the bladder lumen (88% of cases), and invasion of the bladder wall is observed in 92% of adenocarcinomas. Distant metastases are found in 48% of cases.

On MRI, sagittal images are important in defining the location of the tumor. High-intensity focal areas on the T2 sequence are produced by the mucosal component and are strongly suggestive of adenocarcinoma. The solid component is isointense to soft tissue on T1 and improves post-contrast administration.

Besides, slices in sagittal reconstruction by MRI are interesting for specifying the surgical approach.

Cystoscopy with biopsy is the key examination, it allows visualization of the location, size, and degree of invasion as well as the performance of a biopsy. Eighty percent of cases the intravesical part of the tumor, located at the level of the bladder dome [13].

Tumor markers namely CA 125, carbohydrate-antigen 19-9 (CA 19-9), and carcinoembryonic antigen (CEA) are of great interest for post-operative follow-up and monitoring of therapeutic efficacy [14].

As part of an extension assessment, it is imperatively recommended to perform an upper gastrointestinal endoscopy, a colonoscopy and a gynecological examination in search of a primary and/or a secondary location of the adenocarcinoma of the urachus. In our study, all of our patients underwent a complete clinical workup with pelvic examinations and colonoscopy, endoscopy which came back negative.

A work-up combining an upper gastrointestinal endoscopy, colonoscopy, and gynecological examination should be performed to rule out an extension to urachus of colorectal, gastric, or gynecological cancer.

There is currently no effective treatment for this rare disease, surgery is the main treatment option [15]. No definitive evidence regarding the curative effect of chemotherapy and radiotherapy. Various protocols have been studied, some based on cisplatin, others based on fluoro-uracil [15].

The tumor generally progresses towards the extension to neighboring organs (rectal, colic, and uterine) and towards the anterior abdominal wall. Lymph node extension is early, the tumor spreads lymphatically to the iliac, obturator, preaortic and cervical lymph nodes. About 21% of affected patients had distant metastasis at their first presentation. The main metastatic sites are lymph node, pulmonary, bone, and peritoneal. Other sites are also possible, such as the ovaries and peri-rectal soft tissue. The most frequent local recurrences are localized to the pelvis, the bladder, and the abdominal wall [16].

## 3 Conclusion

Urachal adenocarcinoma is a very rare tumor accounting for 0.2–2% of malignant bladder tumors. They affect men twice as much as women. The histogenesis is not yet elucidated, the metaplastic theory is however the most accepted, adenocarcinoma represents the most frequent histological type, 85% of cases. Although the treatment is not well codified but early diagnosis, radical surgery and adjuvant chemotherapy remains the gold standard in the management of this neoplasia.

## Sources of funding

Not applicable.

## Guarantor

Dr. Jandou issam.

## Ethical approval

The study committee of the jura sud hospital center approves the favorable opinion to publish this work.

## Consent to publication

The consent to publish this information was obtained from study participants. We confirm that **written** proof of consent to publish study participants are available when requested and at any time.

## Author's contributions

Dr. IJ, Dr. EA analysed and performed the literature research, Pr. AM, Pr. MD, Pr. AD, Pr. RA performed the examination and performed the scientific validation of the manuscript. Issam Jandou was the major contributors to the writing of the manuscript. All authors read and approved the manuscript.

## Trial registry number

Name of the registry:
Unique Identifying number or registration ID:
Hyperlink to your specific registration (must be publicly accessible and will be checked):

## Availability of data and material

The datasets in this article are available in the repository of the urology database, Chu Ibn-Rochd Casablanca, upon request, from the corresponding author.

## Declaration of competing interest

The authors state that they do not have competing interests.
